# A highly selective KIT inhibitor MOD000001 suppresses IgE-mediated mast cell activation

**DOI:** 10.1016/j.jacig.2024.100249

**Published:** 2024-04-03

**Authors:** Yuki Nakamura, Takeo Urakami, Kayoko Ishimaru, Nguyen Quoc Vuong Tran, Takafumi Shimizu, William Sinko, Taisuke Takahashi, Sivapriya Marappan, Kishore Narayanan, Ramulu Poddutoori, Yoh Terada, Atsuhito Nakao

**Affiliations:** a Department of Immunology, Faculty of Medicine, University of Yamanashi, Yamanashi, Japan; b Alivexis, Inc, Tokyo, Japan; c Aurigene Oncology, Ltd, Bangalore, India; d Yamanashi GLIA Center, University of Yamanashi, Yamanashi, Japan; e Atopy Research Center, Juntendo University School of Medicine, Tokyo, Japan

**Keywords:** KIT inhibitor, mast cells, IgE, allergy

## Abstract

**Background:**

The KIT receptor tyrosine kinase and its ligand, stem cell factor (SCF), control proliferation and survival of mast cells. Thus, targeting KIT signaling may show promise for the treatment of allergic diseases involving mast cells. Recently, we discovered a new compound, MOD000001, as a potential small-molecule KIT kinase inhibitor by using an *in silico* approach.

**Objective:**

We sought to determine whether MOD000001 is highly selective to KIT, inhibits KIT signaling in mast cells, and affects IgE-mediated mast cell activation.

**Methods:**

The interaction of MOD000001 with 468 human kinases and its inhibitory activity against KIT were profiled and evaluated by using KINOMEscan (Discover X/Eurofins Corporation, Fremont, Calif) and cell-free kinase assays, respectively. The effects of MOD000001 on SCF-dependent signaling were examined by using primary mouse and human mast cells. The effects of MOD000001 on SCF-induced degranulation and passive cutaneous anaphylaxis reaction were examined in mice.

**Results:**

MOD000001 interacted with KIT and inhibited KIT kinase activity with high selectivity. MOD000001 suppressed SCF-induced KIT signaling in mouse and human mast cells and in mice. Passive cutaneous anaphylaxis reaction was suppressed in mice treated with MOD000001 both for a short-term (1 week) and for a long-term (7 weeks). Mice treated with MOD000001 for a long-term, but not for a short-term, showed skin mast cell reduction.

**Conclusions:**

MOD000001 is a highly selective KIT inhibitor that can suppress IgE-mediated mast cell activation *in vivo*. MOD000001 may do so by reducing tissue mast cell numbers or by other unknown mechanisms. The findings suggest potential benefits of MOD000001 for allergic diseases involving IgE-mediated mast cell activation.

## Introduction

The KIT (c-KIT/CD117) receptor tyrosine kinase and its only ligand, stem cell factor (SCF), are master regulators of mast cell biology.^1^^,^^2^ Mast cells generate from hematopoietic stem cells, enter circulation as mast cell precursors, and migrate to the tissues, where they mature.^3^ KIT is highly expressed throughout the life of a mast cell. KIT phosphorylation by stroma cell–derived SCF controls differentiation, migration, maturation, survival, and activation of mast cells. For instance, mice deficient in either KIT or SCF lack tissue mast cells,^4^ and imatinib, a KIT kinase inhibitor, reduces mast cell number after long-term dosing in patients with chronic myelogenous leukemia.^5^

Given the crucial importance of mast cells in IgE-dependent and -independent allergic diseases,^4^^,^^6^, ^7^, ^8^ mast cell–targeting strategies, in particular selective KIT inhibition, are an attractive treatment approach for these disorders. Indeed, imatinib and mAbs against KIT ameliorate asthma and chronic urticaria, respectively, likely because of a decrease in mast cell activation and number.^9^^,^^10^ However, imatinib and anti-KIT mAbs typically have limitations (eg, off-target effects on other kinases, immunogenicity, and high production costs). To overcome such disadvantages, we discovered a new compound, MOD000001, as a potentially highly selective small-molecule KIT kinase inhibitor by using an *in silico* approach.^11^^,^^12^

This study aimed to determine whether MOD000001 is indeed highly selective to KIT, inhibits KIT signaling, and affects IgE-mediated mast cell activation.

## Results and discussion

**Fig 1:**
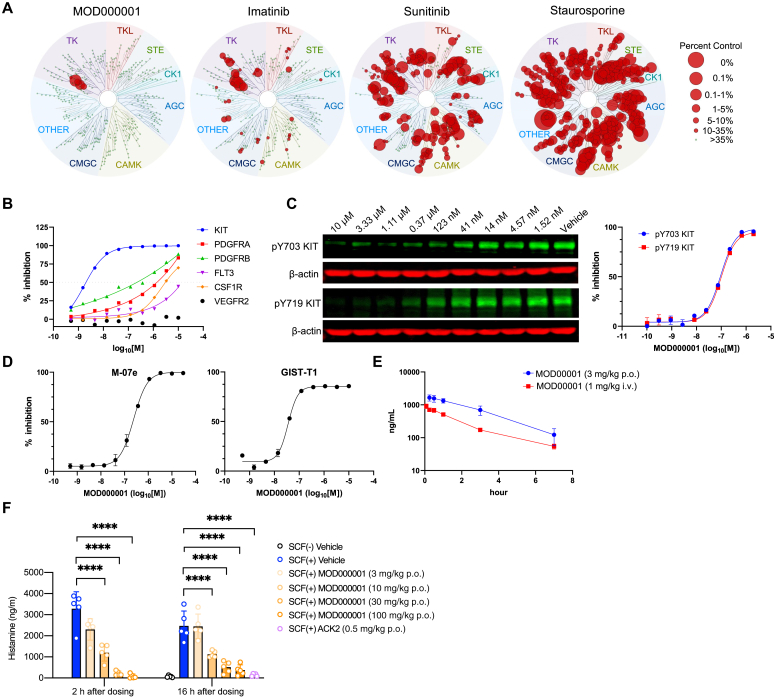
MOD000001 selectively inhibits KIT signaling both *in vitro* and *in vivo*. **A,** Interaction of MOD000001 (3 μM) and other marketed KIT inhibitors (10 μM) with 468 human kinases profiled by KINOMEscan (Discover X/Eurofins). Kinome data of imatinib, sunitinib, and staurosporine were obtained from DiscoverX/Eurofins. Kinases found to bind with the inhibitors are marked with *red circles*, whereas *larger circles* indicate higher-affinity binding. **B,** Cell-free *in vitro* kinase activity of MOD000001 against recombinant human KIT, PDGFRA, PDGFRB, FLT3, CSF1R, and VEGFR2. **C,** KIT phosphorylation assay (tyrosine residues 703 [*top*] and 719 [*bottom*]) in SCF-stimulated NCL2 mouse mast cell line expressing wild-type KIT (*left panel*) and their quantitative analysis (*right panel*). **D,** SCF-dependent growth of M-07e (*left panel*) and GIST-T1 cell (*right panel*) lines. **E,** Plasma levels of MOD000001 analyzed by LC-MS/MS at the indicated hours after oral (3 mg/kg) or intravenous (1 mg/kg, intraperitoneal) administration of the reagent in mice. **F,** Plasma histamine levels at 15 minutes after intravenous injection of SCF in mice. MOD000001 was orally administered 2 hours (*left panel*) or 16 hours (*right panel*) before SCF injection. The group labeled SCF(−) indicated that the mouse group did not receive SCF injection. Data are presented as mean ± SEM (n = 5). Statistical differences were determined by 1-way ANOVA with the Dunnett *post hoc* test. ∗∗∗∗*P* < .0001. *ACG*, Protein kinase A, C, G families; *CAMK*, calcium-/calmodulin-dependent protein kinase family; *CK1*, casein kinase 1 family; *CMGC*, cyclin-dependent kinase (CDK), mitogen-activated protein kinase, GSK3, CDC-like (CLK) families; *GIST*, gastrointestinal stromal tumor; *LC*, liquid chromatography; *MS*, mass spectrometry; *Other*, other kinases; *STE*, homologs of yeast Sterile 7, 11, 20 kinases; *TK*, tyrosine kinase family; *TKL*, tyrosine kinase–like family.

We tested the interaction of MOD000001 with 468 kinases covering more than 80% of the human catalytic protein kinome using KINOMEscan (Discover X/Eurofins Corporation, Fremont, Calif).^13^ MOD000001 showed higher KIT selectivity compared with marketed KIT inhibitors, imatinib and sunitinib,^14^ and a pan-kinase inhibitor, staurosporine (Fig 1, *A*). We then performed cell-free recombinant human kinase assays with KIT and closely related class 3 receptor tyrosine kinases, including platelet derived growth factor-receptor A (PDGFRA) and platelet derived growth factor-receptor B (PDGFRB),^15^ obtaining nanomolar IC_50_ (50% inhibition concentration) values of MOD000001 for KIT (2 nM), but low micromolar or high nanomolar values for PDGFRA and PDGFRB (1400 and 218 nM, respectively) (Fig 1, *B*).

In cell-based assays using the NCL2 mouse mast cell line expressing wild-type KIT, MOD000001 inhibited autophosphorylation of KIT tyrosine residues 703 and 719, which recruit phosphatidylinositol 3-kinase and other signaling proteins (Fig 1, *C*).^15^ MOD000001 also decreased SCF-dependent growth of the M-07e human myeloid leukemia cell line expressing wild-type KIT and the human gastrointestinal stromal tumor T1 cell line expressing constitutively active KIT in a dose-dependent manner (Fig 1, *D*).

To determine whether MOD000001 can inhibit KIT signaling *in vivo*, we examined the effects of orally administered MOD000001 on an SCF-induced degranulation response in rodents.^16^ Pharmacokinetics analysis showed that MOD000001 had 89.7% oral bioavailability with a plasma half-life of approximately 3 hours after a single oral administration of 3 mg/kg MOD000001 in mice (Fig 1, *E*). Oral administration of 3, 10, 30, and 100 mg/kg MOD000001 at either 2 or 16 hours before SCF challenge showed that MOD000001 significantly inhibited SCF-induced plasma histamine increases in a dose-dependent manner in mice (Fig 1, *F*). Thus, MOD000001 can selectively inhibit KIT kinase activity *in vitro* and suppress KIT signaling *in vivo* via the oral route.

**Fig 2:**
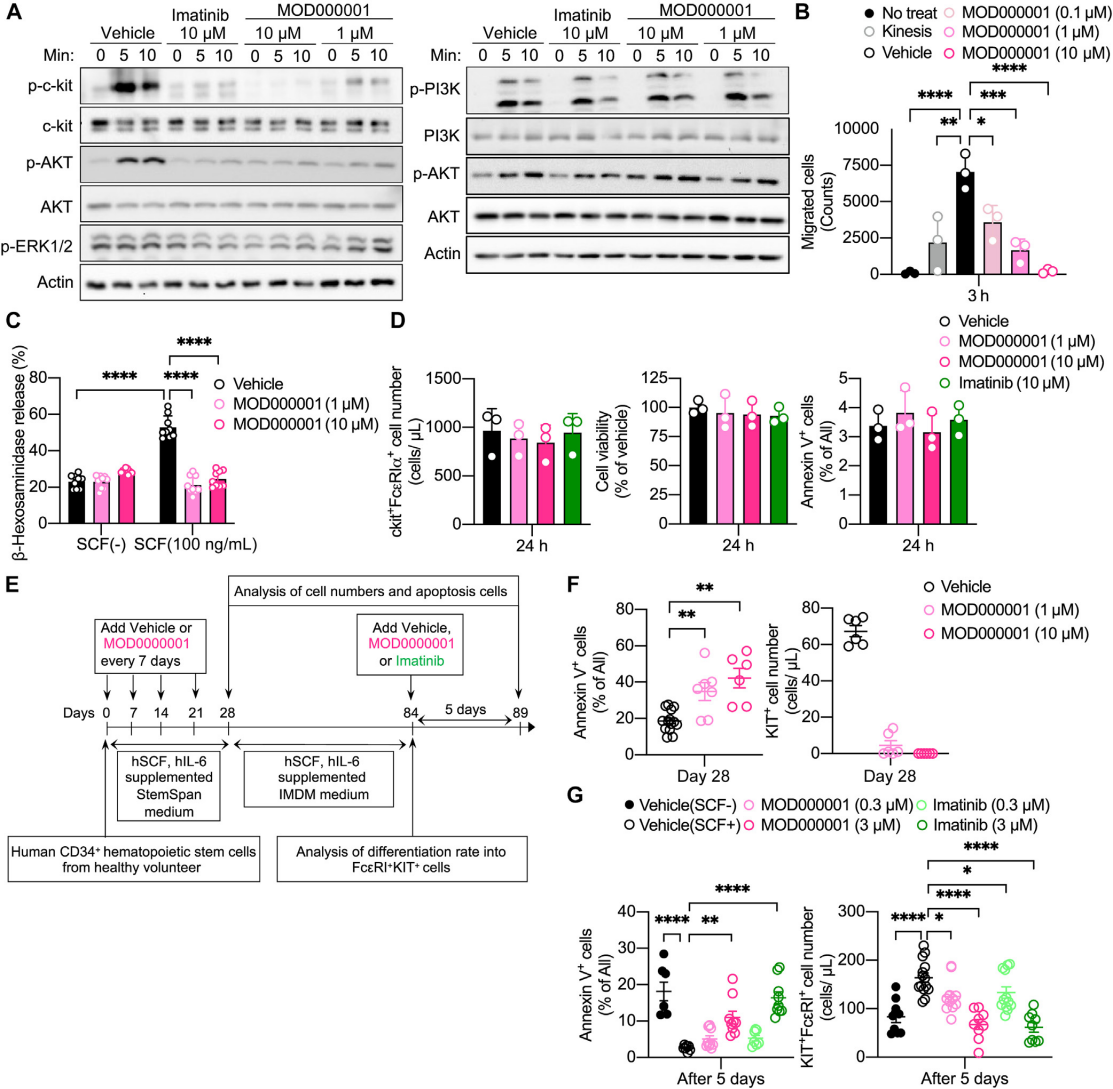
MOD000001 inhibits SCF-/KIT-dependent mouse and human mast cell responses. **A,** SCF-dependent KIT, AKT, and ERK1/2 phosphorylation (*left*) and IgE-dependent PI3K and AKT phosphorylation (*right*) in BMMCs detected by Western blot analysis. **B,** SCF-dependent migration of BMMCs evaluated by Transwell assay (n = 3). **C,** SCF-dependent potentiation of IgE-mediated degranulation in BMMCs (n = 9). **D,** The FcεRⅠα^+^ KIT^+^ cell number, Annexin V^+^ cells, or cell viability was detected by FACS or WST assays after treatment with vehicle, MOD000001, or imatinib for 24 hours (n = 3). **E,** Experimental protocol for human PBMCs. **F,** Annexin V^+^ cells (*left*; n = 7-14) or KIT^+^ cell numbers (*right*; n = 6) at 28 days after culturing human peripheral blood–derived CD34^+^ cells with IL-6 and SCF in the presence of MOD0000001 or vehicle. **G,** Annexin V^+^ cells (*left*; n = 7-9) or FcεRⅠ^+^ KIT^+^ cell numbers (*right*; n = 9-14) of human PBMCs cultured for 5 days in the presence of MOD000001, imatinib, or vehicle. Data are presented as mean ± SD. Statistical differences were determined by 1-way ANOVA with the Dunnett *post hoc* test or 2-way ANOVA with the Tukey *post hoc* test. ∗*P* < .05; ∗∗*P* < .01; ∗∗∗*P* < .001; ∗∗∗∗*P* < .0001. *ERK1/2*, Extracellular signal-regulated kinase 1/2; *FACS*, fluorescence-activated cell sorting; *IMDM*, Iscove's Modified Dulbecco's Medium; *PI3K*, phosphatidylinositol 3-kinase.

We next examined the effects of MOD000001 on SCF-dependent KIT signaling, chemotaxis, and activation in mouse bone marrow–derived cultured mast cells (BMMCs). Treatment of BMMCs with 1 or 10 μM MOD000001 for 1 hour dose-dependently inhibited SCF-dependent KIT autophosphorylation and phosphorylation of AKT and extracellular signal-regulated kinase 1/2 (Fig 2, *A*). In contrast, treatment of BMMCs with 1 or 10 μM MOD000001 for 1 hour did not affect IgE-dependent phosphatidylinositol 3-kinase and AKT phosphorylation (Fig 2, *A*). In addition, treatment of BMMCs with 0.1, 1.0, or 10 μM of MOD000001 for 1 hour inhibited SCF-dependent chemotaxis (Fig 2, *B*).^17^ Low-dose SCF treatment synergistically increased degranulation with allergen-specific IgE.^18^ The SCF-dependent increase in degranulation was inhibited by the pretreatment with 1 and 10 μM of MOD000001 for 1 hour as judged by β-hexosaminidase release assay (Fig 2, *C*). The treatment of BMMCs with 1 or 10 μM of MOD000001 for at least 24 hours did not affect cellular viability as judged by direct cell counting, calorimetric WST assay, and Annexin V staining (Fig 2, *D*). This is likely because BMMCs can survive by depending on IL-3 in the culture medium. These data taken together showed that MOD000001 can inhibit SCF-dependent KIT signaling, chemotaxis, and SCF-induced potentiation of IgE-mediated activation in mouse primary mast cells without affecting cell viability.

We further investigated the effects of MOD000001 on the differentiation of human primary mast cells. CD34^+^ stem cells isolated from human peripheral blood were differentiated into human PBMCs by culturing for 12 weeks (days 0-84) in the presence of IL-6 and SCF (Fig 2, *E*).^19^ The addition of 1 or 10 μM of MOD000001 at day 0 induced apoptosis as determined by Annexin V staining and reduced the number of KIT-positive cells on day 28 (Fig 2, *F*).

We next explored the effects of MOD000001 on the survival of human PBMCs after differentiation (Fig 2, *E*). The addition of 0.3 or 3 μM of MOD000001 into the day 84 culture of human PBMCs induced apoptosis and reduced the number of KIT^+^ FcεRI^+^ cells on day 89 (Fig 2, *G*). Therefore, we concluded that MOD000001 can abrogate SCF-dependent survival and differentiation of human primary mast cells.

**Fig 3:**
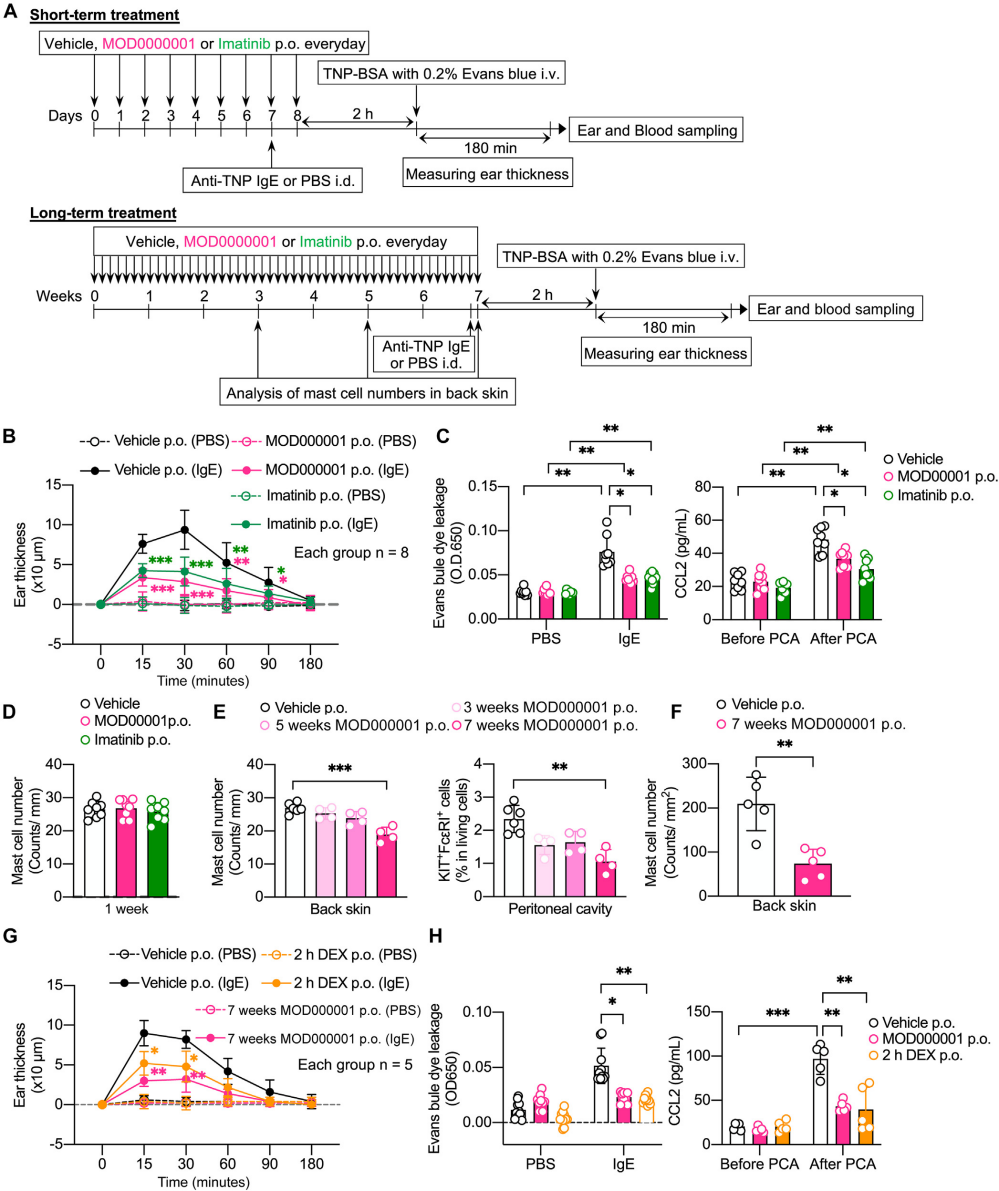
MOD000001 inhibits PCA reaction in mice. **A,** Experimental protocol for PCA reaction (*upper*: short-term treatment; *lower*: long-term treatment). **B,** Kinetics of ear thickness after induction of PCA reaction in mice pretreated with vehicle, MOD000001, or imatinib for 7 days (n = 8). **C,** Evans blue extravasation in the ear (*left*) or serum CCL2 levels (*right*) at 180 minutes after induction of PCA reaction in mice pretreated with vehicle, MOD00001, or imatinib for 7 days (n = 8). **D,** The number of toluidine blue–stained skin mast cells in mice treated with vehicle, MOD000001, or imatinib for 7 days (n = 8). **E,** The number of toluidine blue–stained skin mast cells (*left*) and FcεRⅠ^+^ KIT^+^ peritoneal mast cells (*right*) in mice orally treated with vehicle or MOD000001 for 3, 5, or 7 weeks (n = 4-6). **F,** The number of skin mast cells (n = 5) in Mcpt5-Cre Ai14-TdTomato mice orally treated with vehicle or MOD000001 for 7 weeks after tissue-clearing using the FDISCO+ method. The cleared back skin samples were imaged using a light sheet fluorescence microscope and the number of mast cells was counted by Arivis Vision 4D (please see Video E2). **G,** Kinetics of ear thickness after induction of PCA reaction in mice orally pretreated with vehicle or MOD000001 for 7 weeks (*left panel*) (n = 5). DEX was orally administrated 2 hours before induction of PCA reaction as negative control (n = 5). **H,** Evans blue extravasation in the ear (*left*) or serum CCL2 levels (*right*) at 180 minutes after induction of PCA reaction in mice pretreated with vehicle, MOD00001, or imatinib for 7 weeks (n = 5). Data are presented as mean ± SD. Statistical differences were determined by 1-way ANOVA with the Dunnett *post hoc* test or 2-way ANOVA with the Tukey *post hoc* test. ∗*P* < .05; ∗∗*P* < .01; ∗∗∗*P* < .001; ∗∗∗∗*P* < .0001. *DEX*, Dexamethasone; *TNP*, trinitrophenyl.

Finally, we examined the effects of MOD000001 on the passive cutaneous anaphylaxis (PCA) reaction, a hypersensitivity reaction in the skin (Fig 3, *A*). Imatinib was used for comparison. Oral administration of 100 mg/kg of MOD000001 every day for 7 days inhibited the PCA reaction as well as imatinib, as reflected by decreases in ear thickness, Evans blue dye extravasation, and serum CCL2 levels (Fig 3, *B* and *C*). Notably, toluidine blue staining showed that the mast cell numbers in the mouse skin were comparable between vehicle-treated mice and MOD000001-treated mice (Fig 3, *D*). These results suggest that short-term oral administration (7 days) of MOD000001 can attenuate IgE-mediated mast cell activation in mice.

Inhibition of KIT signaling with anti-KIT mAb rapidly reduces the mast cell burden or plasma tryptase levels in humans approximately 1 week after dosing.^10^^,^^20^ We thus sought to determine how long treatment with MOD000001 was able to affect mast cell numbers in mice (Fig 3, *A*). For this purpose, mice received oral MOD000001 for different treatment durations. Oral administration of 100 mg/kg of MOD000001 for 7 weeks, but not for 3 and 5 weeks, reduced the number of skin and peritoneal mast cells (Fig 3, *E*). In addition, visualization of mast cells using tissue-clearing technology^21^ in Mcpt5-Cre TdTomato^fl/fl^ mice enabled us to capture the reduction of skin mast cells spatially in 3D after a 7-week treatment with 100 mg/kg of MOD000001 (Fig 3, *F*, and Video E1). By using toluidine blue staining, we validated the fluorescence microscope–based findings in Mcpt5-Cre TdTomato^fl/fl^ mice (data not shown). Consistent with these findings, PCA reaction was attenuated in mice orally treated with 100 mg/kg of MOD000001 every day for 7 weeks compared with control mice (Fig 3, *G* and *H*). Thus, long-term oral administration (7 weeks) of MOD000001 can reduce mast cell numbers and attenuate the PCA reaction in mice. It remains to be determined whether there are any compensatory effects of mast cell reduction on other cell types or whether the effects of MOD000001 on the mast cell numbers are persistent after MOD000001 is stopped.

It is unclear how short-term (7 days) oral MOD000001 attenuated the PCA reaction without altering the number of skin mast cells (Fig 3, *B* and *C*); however, the PCA reaction is likely to occur with a background of SCF-mediated KIT activation *in vivo*. We thus speculate that stroma cells in the skin tissue may continuously provide SCF to mast cells *in vivo*, and inhibition of SCF/KIT signaling by MOD000001 could reduce the severity of the PCA reaction, as evidenced by the *in vitro* inhibition of SCF-dependent potentiation in IgE-mediated degranulation after 1-hour treatment with MOD000001 (Fig 2, *C*). Similar to our findings, a single dose of imatinib or nilotinib, a second-generation tyrosine kinase inhibitor, by oral gavages attenuated a mouse model of asthma and PCA reaction just approximately 1 hour before allergen challenge in rodents.^22^^,^^23^

It also remains to be determined how MOD000001 reduced tissue mast cell numbers in mice when dosed long-term (7 weeks) (Fig 3, *E* and *F*). This might be due to the months-long lifespan of tissue mast cells in mice, possibly depending on compensatory IL-3 (or other cytokines) in the aberrant conditions wherein KIT signaling was blocked by MOD000001. Thus, the reduction of mast cell numbers might be observed when the lifespan of IL-3 (or other cytokines)–dependent tissue mast cells ended and MOD000001 could inhibit SCF-dependent differentiation/survival, migration into the skin, or *in situ* maturation of newly recruited mast cell precursors. This is in contrast to the rapid reduction of human skin mast cells after the treatment with anti-KIT mAb for 1 week or more,^10^^,^^20^ suggesting that human mast cells predominantly depend on SCF/KIT signaling for their maintenance compared with mouse mast cells, which we showed using human and mouse primary mast cells. Therefore, the effective doses of MOD000001 could be reduced in human mast cells compared with those in mouse mast cells.

We showed that MOD000001 can ameliorate IgE-mediated mast cell activation by reducing tissue mast cell numbers or by other unknown mechanisms. The effective doses of MOD000001 and other KIT inhibitors such as imatinib appeared to be largely comparable. However, given its high selectivity to KIT compared with other KIT inhibitors, MOD000001 may be a good drug candidate for the treatment of allergic diseases in terms of avoidance of potential adverse effects. Although this specific compound would need to be further optimized for the clinic, we believe that a highly selective KIT inhibitor similar to MOD000001 might have potential benefit for the treatment of allergic diseases that involve IgE-mediated mast cell activation.

## Disclosure statement

This research was funded by a grant-in-aid for scientific research granted to A.N. from the 10.13039/501100001700Ministry of Education, Culture, Sports, Science and Technology, Japan (grant no. 22K19427). A.N. also received a research grant from Alivexis, Inc.

Disclosure of potential conflict of interest: The authors declare that they have no relevant conflicts of interest.
