# Supplementary material for: A highly selective KIT inhibitor MOD000001 suppresses IgE-mediated mast cell activation
Source: J Allergy Clin Immunol Glob. 2024 Apr 3;3(3):100249. doi: 10.1016/j.jacig.2024.100249 (PMC11101940; doi:10.1016/j.jacig.2024.100249)
Supplement: Supplementary Data [file mmc3.docx]

**Online Repository (Methods)**

**MOD000001**

MOD000001 was discovered as a small molecule KIT inhibitor by Modulus Discovery Inc. (Tokyo, Japan), synthesized at Aurigene Oncology, Inc (Bangalore, India), and used for the experiments described below.^E1, E2^

**Interaction of MOD000001 with 468 human kinases**

The KINOMEscan^®^ screening platform is a site-directed competition binding assay to quantitatively measure interactions between test compounds and 468 kinases and disease-relevant mutant variants (Eurofins DiscoverX, San Diego, CA).^E3^ The selectivity of MOD000001 against 468 human kinases was profiled at a concentration of 3 µM in the scanMAX Kinase Assay Panel (Eurofins DiscoverX, San Diego, CA) following the instruction by the company. The results for the binding interactions are reported as "% control (Ctrl)", where lower numbers indicate stronger hits in the matrix as follows;

%Ctrl Calculation

test compound signal - positive control signal

negative control signal - positive control signal

x 100

Test compound = MOD000001 (3 μM)
Negative control = DMSO (100%Ctrl)
Positive control = control compound (0%Ctrl)

Images were generated using TREEspot™ Software Tool and were reprinted with permission from KINOMEscan®, a division of DiscoveRx Corporation, © DiscoverX Corporation.

Abbreviation in Fig. 1A are as follows;

ACG: Protein kinase A, C, G families, CAMK: calcium/calmodulin-dependent protein kinase family, CK1: casein kinase 1 family, CMGC: cyclin-dependent kinase [CDK]、MAPK, GSK3, CDC-like [CLK] families, STE: homologs of yeast Sterile 7, 11, 20 kinases, TK: tyrosine kinase family, TKL: tyrosine kinase-like family. Others: other kinases.

***In vitro* kinase assay**

The inhibitory activity of MOD000001 against recombinant human KIT, PDGFRA/B, FLT3, CSF1R, and VEGFR2 was evaluated by ^33^P-ATP-based kinase assay (HotSpot™ assay) at Reaction Biology Corporation (Malvern, PA).

**Cell lines**

Human megakaryoblast cell line M-07e cells were purchased from AcceGen Biotech (Fairfield, NJ) and cultured in complete medium, RPMI 1640 medium supplemented with 20% FBS and 50 ng/mL rhSCF (#255-SC, R&D Systems, Minneapolis, MN). GIST-T1 cells were purchased from Cosmo Bio (Tokyo, Japan) and cultured in DMEM supplemented with 10% FBS. NCL-2 cells were purchased from Applied Biological Materials Inc. (Richmond, BC) and cultured in Minimum Essential Medium α (MEMα) supplemented with 10% FBS, 50 μM 2-mercaptoethanol, 2 mM l-glutamine, and 5 ng/ml IL-3.

**KIT phosphorylation assay**

Three million M-07e cell were seeded with 3 mL of supplement-free RPMI 1640 medium and treated with inhibitor for 2 hours at 37°C. Cells were stimulated with 50 ng/mL rhSCF for 5 minutes and lysed with Cell Lysis buffer. Site-specific phosphorylated KIT protein was quantified by PathScan® Phospho-c-Kit Sandwich ELISA KIT with phospho-c-Kit Y719 (#7298, Cell Signaling Technology, Danvers, MA) modified with the phospho-c-Kit Y703 antibody (#AF3152, Affinity Biosciences, China) for the detection of phosphorylation at Y703 KIT. Ten million NCL-2 cells were preconditioned in the supplement-free media and incubated at 37°C and 5% CO2 for 2　hours. The cells were then treated with MOD000001 for 2 hours and stimulated with 25 ng/ml of rmSCF (#455-MC, R&D systems) for 10 minutes. Cells were lysed with Cell Lysis buffer and phosphorylated KIT were detected by anti-pKIT Y703 antibody (AF3152) or anti-pKIT Y719 antibody (#3391, Cell Signaling Technology).

**Cell proliferation assay**

M-07e cells (5000 cells/well) and GIST-T1 cells (2000 cells/well) were plated on 96-well cell culture plates with complete medium and treated with MOD000001 at different concentrations. Cells were cultured for 72 hours and quantified by CellTiter-Glo® Reagent (Promega, Madison, WI). The luminescence at the time of plating cells was used as the Day 0 control and IC50 values were determined by Prism9 software (GraphPad Software, Boston, MA).

**Mice**

Female 8- to 9-week-old BALB/c mice were purchased from Vivo Bio Tech (Hyderabad, India). Male 8- to 10-week-old C57BL/6 mice (Japan SLC, Tokyo, Japan), *Mcpt5*-Cre transgenic mice^E4^ and Ai14 mice (Strain #007914, The Jackson Laboratory, USA) were bred under specific pathogen-free conditions. *Mcpt5*-Cre transgenic mice express Cre recombinase under the control of the mast cell protease (Mcpt) 5 promoter in the connective tissue-type mast cells. Ai14-tdTomato mice have a loxP-flanked STOP cassette preventing transcription of a CAG promoter-driven red fluorescent protein variant (tdTomato), which express robust tdTomato fluorescence following Cre-mediated recombination. *Mcpt5*-Cre mice were mated with Ai14-tdTomato mice in our laboratory to generate double-transgenic *Mcpt5*-Cre Ai14-tdTomato mice, which have high expression of tdTomato in *Mcpt5*-expressing mast cells. All mice were housed for at least two weeks under 12-hour light/12-hours dark conditions with *ad libitum* access to food and water. All animal experiments were approved by the Institutional Animal Ethics Committee of Aurigene Oncology and the Institutional Review Board of the University of Yamanashi (No. A3-40).

**Pharmacokinetics analysis**

For the intravenous injection, MOD000001 was dissolved at 0.1 mg/mL in vehicle (2% *N*-methylpyrrolidone, 30% PEG-200 and 68% Saline) and dosed at the volume of 10 mL/kg body weight via the tail vein. For the oral gavage, MOD000001 was suspended in 0.5% Tween 80 and 0.5% methylcellulose solution (oral gavage vehicle). Blood was collected periodically from the saphenous vein and centrifuged to obtain plasma samples. Plasma protein was removed by acetonitrile precipitation method and concentration of MOD000001 was analyzed by LC-MS/MS with Nexera X2 (Shimadzu) and API 4000 (Applied Biosystems).

**Systemic anaphylaxis model**

Female BALB/c mice were used for experiments at 8-9 weeks of age. MOD000001 was suspended in the oral gavage vehicle and prepared at concentrations of 0.3, 1, 3 and 10 mg/mL. Mice were orally administered oral gavage vehicle or MOD000001 suspension at volumes of 10 mL/kg body weight at 2 or 16 hours before the recombinant mouse SCF (rmSCF) injection. For the induction of systemic anaphylaxis, mice were injected with either PBS or 100 µg/kg rmSCF (#455-MC, R&D Systems) intravenously via the tail vein. Fifteen minutes after the rmSCF injection, mice were anesthetized with isoflurane and blood was collected from a large vein into a tube with K_2_EDTA. Blood samples were centrifuged at 4°C and plasma samples were collected and stored at -80°C until histamine analysis. Plasma histamine concentrations were measured by Histamine EIA enzyme KIT (#EA31, Oxford Biomedical Research, Rochester Hills, MI) following manufacture’s instruction.

**Preparation of mouse bone marrow-derived mast cells (BMMCs)**

Bone marrow-derived mast cells (BMMCs) were generated from the femoral bone marrow cells of male mice as previously described.^E5^ Briefly, whole bone marrow cells were cultured in RPMI 1640 supplemented with 10% fetal bovine serum, 2 mM L-glutamine, 10 mM nonessential amino acids, penicillin/streptomycin, 10 mM sodium pyruvate, and 50 μM 2-ME (complete RPMI1640) in the presence of 10 ng/ml recombinant mouse IL-3 (rmIL-3). Only floating cells were sub-cultured twice per week, and further expanded for 4-6 weeks in fresh complete RPMI 1640 supplied with rmIL-3. Finally, the cells (>95% FcεR1^+^KIT^+^) were used as BMMCs without further purification.

**Flow cytometric analysis (FACS)**

Mouse BMMCs were stained with antibodies specific for PE-conjugated anti-mouse c-kit (clone;2B8, BioLegend, USA) and FITC-conjugated anti-mouse FcεRI (clone;MAR-1, BioLegend, USA) in the presence of anti-mouse CD16/CD32 (Mouse BD Fc Block™, BioLegend, USA). Human CD34^+^ cells and human peripheral blood-derived mast cells (huPBMCs) were stained with antibodies specific for PE-conjugated anti-human CD34 (Miltenyi Biotec, USA), FITC-conjugated anti-human CD45 (Miltenyi Biotec, USA), PE-conjugated anti-human CD117(c-kit) (clone;104D2, BioLegend, USA) or FITC-conjugated anti-human FcεRI (clone; AER-37, BioLegend, USA) in the presence of human FcR blocking reagent (Miltenyi Biotec, USA). After washing with PBS, the stained cells were analyzed on a BD Accuri C6 flow cytometer (BD Biosciences). For detection of apoptotic cells, BMMCs and HuPBMCs were incubated with 5 μl FITC–conjugated annexin V and 2.5 μl propidium iodide (PI) in 50 μl of 1X binding buffer for 15 min. After adding 200 μl of 1X binding buffer, the stained cells were analyzed on the flow cytometer. Flow cytometry data were analyzed using the BD Accuri C6 software (BD Biosciences).

**Cell viability**

Cell viability was assessed using the calorimetric water-soluble tetrazolium salt (WST) assay, direct cell counting, and Annexin V staining. For the WST assay, BMMCs treated with 1 or 10 μM MOD000001 or imatinib were subjected to the WST8 assay (Cell Counting Kit-8, DOJINDO LABORATORIES, Kumamoto, Japan), following the manufacturer’s instructions. For direct viable cell counting, BMMCs (1 × 10^6^ cells/ mL) were treated with 1 or 10 μM MOD000001 or imatinib for 24 hours, and the number of BMMCs was subsequently counted by a hemacytometer. For apoptosis cells detection, the BMMCs (1 × 10^6^ cells/ mL) were treated with 1 or 10 μM MOD000001 or imatinib for 24 hours and were evaluated by FACS using the FITC conjugated-Annexin V Apoptosis Detection Kit (DOJINDO LABORATORIES, Kumamoto, Japan), following the manufacturer’s instructions. The human CD34^+^ cells were treated with 1 or 10 μM MOD000001 from the beginning of the culture. The mature huPBMCs were treated with 0.3 or 3 μM MOD000001 or imatinib for 5 days. After each treatment, cell viability was assessed by the above methods.

**Western blot**

SCF (100 ng/mL)-induced KIT, AKT and Erk1/2 phosphorylation or anti IgE (1μg/mL)-induced PI3K and AKT phosphorylation in MOD000001 (1 or 10 μM) or Imatinib (10 μM) 1 hour pretreated BMMCs were detected by Western blotting as previously described.^E6^ Briefly, BMMCs were lysed in RIPA buffer (25 mM Tris-HCl, pH7.6, 150 mM NaCl, 1% Triton X100, 1% sodium deoxycholate, 0.1% SDS) with protease inhibitor cocktail (Merck Millipore, Burlington, MA) and vanadate (FUJIFILM Wako Pure Chemical Corporation, Osaka, Japan). Cell lysates were dissolved in sample buffer containing 50 mM dithiothreitol and bromophenol blue, and then boiled for 5 minutes. Protein concentrations were measured on a NanoDrop ND-1000 (Thermo Fisher Scientific). Proteins were subjected to SDS-PAGE gels and transferred to polyvinylidene fluoride membranes. Blots were immersed in 5% milk blocking solution for 1 hour at room temperature (RT), followed by incubation with anti-phospho c-kit (Try719) (1/1000 dilution), anti-phospho AKT(D9E) (1/2000 dilution), anti-phospho Erk1/2 (Thr202/Tyr204) (1/1000 dilution), anti-phospho PI3K (p85[Tyr458]/p55[Tyr199]) (1/1000 dilution), anti-KIT (D13A2) (1/1000 dilution), anti-AKT (Ser473) (1/1000 dilution), anti-PI3K p85 (1/1000 dilution) and anti-β-actin (13E5) (1/1000 dilution) solution overnight at 4°C. Membranes were washed three times with TBS/T, and then incubated in an anti-rabbit IgG, HRP-linked antibody (1/2000 dilution) solution for 40 minutes at room temperature. Immunoreactive proteins were visualized using ECL Prime (GE Healthcare). All antibodies were purchased from Cell Singling Technology, Inc.

**Chemotaxis**

Chemotaxis was determined by using Transwell polycarbonate membranes (8-μm pore size; Corning, Corning, NY) as previously described.^E7^ Briefly, BMMCs (1 × 10^5^ cells) were incubated in complete RPMI1640 medium for 4 hours and then resuspended in 100 μL of complete RPMI1640 medium and placed in the upper chamber over the bottom chamber containing 600 μL of complete RPMI1640 medium for 30 minutes at 37°C. After 30 minutes, the inserts were placed in chambers containing media with or without SCF (30 ng/mL). After 4 hours, migrated cells were collected from the bottom chamber and counted by using microscopy.

**β-hexosaminidase release assay**

β-hexosaminidase release assay was performed to evaluate the inhibitory effect of MOD000001 on mast cell degranulation *in vitro* as previously described.^E5^ Briefly, IgE (1 μg/mL)-sensitized BMMCs were treated with MOD000001 (1 or 10 μM) for 1 hour before anti-IgE (1 μg/mL) with or without SCF (100ng/mL) stimulation for 40 minutes at 37°C. Total release sample was obtained by adding 1% Triton buffer. The supernatants were collected from each well and mixed with p-nitrophenyl-N-acetyl-β-D-glucosaminide (Sigma, USA) to determine the enzymatic activity of the released β-hexosaminidase. After 90 minutes at 37°C, the reaction was stopped by adding 0.2 M glycine solution, and measured by absorption spectrometer with 405 nm filter. The percentage of β-hexosaminidase release was calculated as follows:

β-hexosaminidase release (%) = OD of stimulated supernatant/OD of supernatant of Triton-lysed cellsX100

**Human peripheral blood cell-derived mast cells (huPBMCs) generation**

Primary huPBMCs were generated as previously described with a little modification.^E8,9^ Briefly, the multipotent CD34^+^ progenitors were isolated from peripheral blood from healthy volunteers after written informed consent under the protocol (No. 2504) agreed upon by the Institutional Review Board of the University of Yamanashi using CD34 Microbead Kit UltraPure (Mitenyi Biotech, Germany). The isolated CD34^+^ cells were cultured using StemSpan medium (STEMCELL Technologies, CANADA) supplemented with 50 ng/mL recombinant human IL-6, 100 ng/mL recombinant human SCF (Peprotech, USA ) and 1% antibiotic/antimycotic mixed solution (Nacarai, Japan) for 4 weeks with or without DMSO (Vehicle) or MOD000001 (1 or 10 μM). After 4 weeks, the vehicle treated cells were cultured using IMDM, GlutaMAX™ Supplement medium（Gibco^TM^ Thermo Fisher, USA）supplemented with 0.5% of BSA (Fuji film WAKO, Japan), 1% Insulin-transferrin selenium (Gibco^TM^ Thermo Fisher, USA), hIL-6 (50 ng/mL), hSCF (100 ng/mL) and 1% antibiotic/antimycotic mixed solution (Nacarai, Japan) for 12 weeks. The cells differentiation rate into mast cells (CD117^+^/FcεRⅠ^+^) was evaluated by FACS with PE anti-human CD117 (KIT) antibody [Clone:104D2] and FITC anti-human FcεRIα antibody [Clone:AER-37] (BioLegend, USA). Cells over 90% differentiated into CD117^+^/FcεRⅠ^+^ cells were used for experiments. This study using human mast cells was approved by the Institutional Ethics Committee of University of Yamanashi, Faculty of Medicine (No. 2504).

**PCA reaction**

PCA reaction was performed to evaluate the inhibitory effect of orally administrated MOD000001 on mast cell degranulation in mice as previously described.^E10^ Briefly, sera from naive mice were obtained 1 week before the beginning of the PCA as control. One week, 7 weeks MOD000001 (100mg/kg p.o.) or 2 hours Dexamethasone (DEX) (10 mg/kg p.o.) treated mice were passively sensitized with IgE by intradermal injection (i.d.) of mouse anti–TNP IgE (10 ng/10 μL/ear, clone; C38-2, BD Pharmagen, USA) in the left ear. The same volume of vehicle (PBS) was injected into right ear as a control. After 24 hours, IgE i.d., the mice were i.v. injected with TNP-BSA (50 μg/200 μL/mouse, LSL, Japan) solution with 0.2% Evans blue dye using the 29G BD ultra-fine insulin syringe (Becton dickinson, USA). Ear thickness was measured immediately before (0 minutes) and 15, 30, 60, 90, and 180 minutes after TNP-BSA challenge using a dial thickness measuring tool (OZAKI MFG.CO.,LTD., Japan). The increase in ear thickness (Δ ear thickness) was calculated by subtracting the values of 0 minutes ear thickness from each time point of ear thickness. Evans blue dye extravasation in ear was evaluated by collecting ear tissue 180 minutes after PCA induction. The ear samples were incubated in 500 μL N,N-Dimethylformamide (TCI, Japan) for 3 hours at 55°C, and the supernatants were collected after centrifugation at 15,000 rpm at room temperature. Evans blue density in the supernatant was measured by absorption spectrometer with a 620 nm filter. To evaluate MCP-1 levels, serum was collected 180 minutes after TNP-BSA i.v. administration.

**Enzyme-linked immunosorbent assay (ELISA)**

The concentrations of MCP-1 (CCL2) in the sera were determined by ELISA. Kits for mouse CCL2/JE/MCP-1 (R & D systems, Minneapolis, MN) were obtained from the indicated suppliers.

**Toluidine blue staining**

Mast cell numbers in the ear after 1, 3, 5, and 7 weeks treatment of MOD000001(100mg/kg p.o. every day) were evaluated by toluidine blue staining. The collected ears were fixed with 4% paraformaldehyde and paraffin-embedded, after which 10 μm sections were prepared. The sliced ears were stained with 0.1% Toluidine blue (pH 4.1). The numbers of mast cells in the ears were quantified by microscopy.

**Peritoneal mast cells**

Mast cell numbers in the peritoneal cavity after 1, 3, 5, and 7 weeks treatment of MOD000001 (100 mg/kg p.o. every day) were evaluated by FACS with anti-KIT and anti-FcεRⅠα Ab. Peritoneal mast cells were collected as previously described.^E5^ Briefly, peritoneal mast cells were collected by injection of cold PBS with 0.5% FBS into the peritoneal cavity, and the abdomen was gently massaged for 60 seconds and all following steps were conducted at 4°C.

**Chemicals and reagents for tissue clearing**

For tissue clearing, we used the following chemicals: tetrahydrofuran with 250 ppm BHT as inhibitor (THF) (186562-1L, Sigma-Aldrich), dibenzyl ether (DBE) (108014, Sigma-Aldrich), N,N,N′,N′-tetrakis (2-hydroxypropyl) ethylenediamine (T0781, Tokyo Chemical Industry Co., Ltd.), triethylamine (34805-75, Nacalai Tesque), and Ethyl cinnamate (051-00792, FUJIFILM Wako Pure Chemical Corporation).

**Tissue clearing (skin)**

The tissue clearing method was performed according to the FDISCO^+^ method^E12^ with some modifications. Briefly, the back skins of mice were fixed overnight at 4°C in 4% PFA followed by 1 hour at room temperature. After fixing, samples were washed with 30 mL PBS in a 50 mL tube to remove PFA for 2 hours 3 times at room temperature. The last washing step was performed overnight. Samples were delipidated by immersion in 50% THF (10 mL THF and 10 mL ddH2O with 20 μL triethylamine to adjust pH to about 9.0) overnight. Then, samples were dehydrated by subsequently immersed in 70% THF (14 mL THF and 6 mL ddH2O with 30 μL triethylamine) for 1 hour, then 80% THF (16 mL THF and 4 mL ddH2O with 50 μL triethylamine) for 1 hour, then 100% THF (with 100 μL triethylamine) for 1 hour twice. All THF solutions were cooled down at 4^o^C for 1 hour before being used. All the delipidation and dehydration steps were performed at 4^o^C with light shaking. The pH of THF solutions was determined using a pH strip.

Clearing solution was prepared by adding 0.5% (w/v) N,N,N′,N′-tetrakis (2-hydroxypropyl) ethylenediamine to the DBE. Samples were immersed in 10 mL of clearing solution at room temperature until clear, then replaced with fresh clearing solution, protected from light, and stored at room temperature. All steps were performed using tight capped glass vials (5-115-06 and 5-115-08, As One Cooperation, Osaka, Japan). All the cleared samples were imaged within 3 days after clearing.

**Light sheet fluorescence microscopy and Imaging data processing**

Samples were imaged using a light sheet fluorescence microscope (Lightsheet 7, Zeiss, Oberkochen, Germany). The cleared tissues were mounted on the sample holder and incubated with Ethyl cinnamate in the sample reservoir. The imaging parameters were kept the same between samples. In particular, laser intensity and exposure time were kept in the range of 20-25% and 10-12.5 millisecond, respectively. Z-step interval in the range of 4 to 5 μm was used. The obtained images were analyzed by Arivis Vision 4D software (Carl Zeiss Microscopy Software Center, Rostock, Germany). tdTomato^+^ cells were detected by applying the “Detect Cells or Particles” in the analysis pipeline of Arivis Vision 4D. Firstly, the diameter and volume of cells were manually measured to determine the range of diameter, volume, and fluorescence intensity. Then, the region of interest was determined, and objects with diameter in the range were detected, then filtered by volume, sphericity, and fluorescence intensity. 3D-rendered images and movies were visualized and captured with Arivis Vision 4D.

**Statistics**

Data analysis was performed using GraphPad Prism 8 (GraphPad software Inc., MA). Results are expressed as mean ± SD and the “n” numbers for each dataset are provided in the figure legends. Statistical significance was assessed by two-tailed Student’s *t* test (Fig 3F), one-way followed by Dunnett’s (Fig. 1F or 3E), Tukey’s post-hoc test (Fig 2B, 2F or 2G) or two-way ANOVA followed by Tukey’s (Fig 2C, 3B, 3C or 3H). Mean ± SEM was shown in Fig 1. Mean ± SD was shown in Fig 2 and 3. *P* values <0.05 (shown as *P<0.05, **P<0.01, ***P<0.001 or ****P<0.0001) were considered significant.

**References for the METHODS**

E1. Takahashi T, Shimizu T, Terada Y and Urakami T. KIT Inhibitors, Compounds, Pharmaceutical Compositions, and Methods of Use Thereof. International Application Number: PCT/JP2023/027945

E2. Urakami T, Takahashi T, Shimizu T, Sinko W, Nakamura Y, Ishimaru K,

Tran VNG, Podutoori R, Narayanan K, Marappan S, Nakao A, Terada Y. Identification of MOD000001, a novel highly selective and orally available KIT inhibitor designed for treatment of mast cell-associated disorders. J Allergy Clin Immunol 151, issue 2, Supplement, AB204

E3. Davis MI, Hunt JP, Herrgard S, Ciceri P, Wodicka LM, Pallares G, Hocker M, Treiber DK, Zarrinkar PP. Comprehensive analysis of kinase inhibitor selectivity. Nat Biotechnol. 2011;29:1046-1051.

# E4. Scholten J, Hartmann K, Gerbaulet A, Krieg T, Müller W, Testa G, Roers A. Mast cell-specific Cre/loxP-mediated recombination in vivo. Transgenic Res 2008;17:307–315.

# E5. Nakamura Y, Nakano N, Ishimaru K, Ando N, Katoh R, Suzuki-Inoue K, Koyanagki S, Ogawa H, Okumura K, Shibata S, Nakao A. Inhibition of IgE-mediated allergic reactions by pharmacologically targeting the circadian clock. J Allergy Clin Immunol. 2016,137:1226-1235.

E6. Nakajima S, Ishimaru K, Kobayashi A, Yu G, Nakamura Y, Oh-Oka K, Suzuki-Inoue K, Kono K, Nakao A. Resveratrol inhibits IL-33-mediated mast cell activation by targeting the MK2/3-PI3K/Akt axis. Sci Rep 2019;9:18423.

E7. Meininger CJ, Yano H, Rottapel R, Bernstein A, Zsebo KM, Zetter BR

[The c-kit receptor ligand functions as a mast cell chemoattractant.](https://pubmed.ncbi.nlm.nih.gov/1371080/)

Blood. 1992;79:958-63.

E8. Saito H, Kato A, Matsumoto K, Okayama Y. Culture of human mast cells from peripheral blood progenitors. Nature protocols 2006;1:2178

E9. Joulia R, Gaudenzio N, Rodrigues M, Lopez J, Blanchard N, Valitutti S, Espinosa E. Mast cells form antibody-dependent degranulatory synapse for dedicated secretion and defence. Nat Commun 2015;6:6174,

# E10. Bahri R, Custovic A, Korosec P, Tsoumani M, Barron M, Wu J, Sayers R, Weimann A, Ruiz-Garcia M, Patel N, Robb A, Shamji MH, Fontanella S, Silar M, Mills ENC, Simpson A, Turner PJ, Bulfone-Paus S. Mast cell activation test in the diagnosis of allergic disease and anaphylaxis. J Allergy Clin Immunol 2018;142:485-96.

E11. Matsushita K, Li X, Nakamura Y, Dong D, Mukai K, Tsai M, Montgomery SB, Galli SJ. The role of Sp140 revealed in IgE and mast cell responses in Collaborative Cross mice. JCI Insight 2021;6:e146572.

E12. Wan P, Li Y, Zhu J, Xu J, Liu X, Yu T, Zhu D. FDISCO+: a clearing method for robust fluorescence preservation of cleared samples. Neurophotonics. 2021;8:035007.

**Video Legend:**

**Movie 1. Representative 3D-rendered image of the skin mast cells in a *Mcpt5*-Cre Ai14-tdTomato mouse orally treated with vehicle for 7 weeks after tissue-clearing using FDISCO+ method**

The cleared back skin samples were imaged using a light sheet fluorescence microscope. The skin mast cells, identified as tdTomato^+^ cells (red), were detected by applying the “Detect Cells or Particles” in the analysis pipeline of Arivis Vision 4D (analysis condition details as described in the Method section). Detected mast cells were marked light blue after one rotation of the image. The skin mast cell numbers in Fig. 3F were counted only light blue colored objects. Some rod-shaped (top region) or zonal (bottom region) fluorescence signals are non-specific autofluorescence signals made by intradermal hair or muscle layer, respectively. The scale is shown in the movie along with a 3D-rendered skin tissue image, with x-, y- and z-axis total length each displayed as 2100μm, respectively.

**Movie 2. Representative 3D-rendered image of the skin mast cells in a *Mcpt5*-Cre Ai14-tdTomato mouse orally treated with MOD000001 for 7 weeks after tissue-clearing using FDISCO+ method**

The cleared back skin samples were imaged using a light sheet fluorescence micro scope. The skin mast cells as tdTomato^+^ cells (red) were detected by applying the “Detect Cells or Particles” in the analysis pipeline of Arivis Vision 4D (detail of the analysis condition were described in the Method section). The detected mast cells were marked light blue after one rotation of the image. The skin mast cell numbers in Fig. 3F were counted only light blue colored objects. Some of the rod-shaped (top region) or zonal (bottom region) fluorescence are non-specific autofluorescence signals by intradermal hair or muscle layer, respectively. Scale was shown in the movie along with 3D-renderd skin tissue image which x-, y- and z-axis total length are displayed in 2100μm, respectively.
